# Evaluation of low-viscosity bulk-fill composites regarding marginal and internal adaptation

**DOI:** 10.1007/s10266-020-00531-x

**Published:** 2020-06-09

**Authors:** Kyung-Jin Park, Manon Pfeffer, Thomas Näke, Hartmut Schneider, Dirk Ziebolz, Rainer Haak

**Affiliations:** Department of Cariology, Endodontology and Periodontology, University of Leipzig, Liebigstr.12, 04103 Leipzig, Germany

**Keywords:** Bulk-fill composite, Low-viscosity bulk-filll composite, High-viscosity bulk-fill composite, Marginal gap formation, Internal adhesive defect

## Abstract

This study aimed at evaluating the marginal and internal adaptation of low-viscosity bulk-fill composites to enamel and dentin using a self-etch or an etch-and-rinse adhesive without and with artificial ageing. Hundred and twenty-eight MOD cavities in extracted molars were assigned to eight groups (*n* = 16), restored with the adhesives OptiBond FL (OFL) or Xeno V+ (X) and two low-viscosity bulk-fill composites SDR or x-tra base, covered with Premise. Tetric EvoCeram Bulk Fill and Premise served as a control. *n* = 8 per group were subjected to prolonged water storage (180 days) and thermocycling (2500×). Scanning electron microscopy was used to determine marginal gaps (MG) and interfacial adhesive defects (IAD). There were no significant differences between composite types in 44 out of 48 (MG) or 43/48 (IAD) comparisons. More MG were observed with X than with OFL (14 out of 16 comparisons, two significant), while in 16 of 16 comparisons with X more IAD were observed (14 significant). After artificial ageing, MG generally increased (9/16 significant), compared to IAD (one significant). The performance of the investigated composite types concerning the integrity of the tooth-composites interface was comparable. Compared to the 1-step self-etch system, the bond with the 3-step etch-and-rinse adhesive was raised.

## Introduction

Since the end of the 1990s, due to improved durability and stability, the number of clinical indications for composite materials has grown and usage has continuously increased [1]. Today composite materials are the primary choice for direct restorations in the dental practice and clinical studies report positive outcomes of resin composites with increased longevity [2–5]. Since the year 2000 developments in resin composites are more focused on systems with reduced polymerization shrinkage and shrinkage stress to prevent consequential failures such as adhesive defects, postoperative sensitivity and restoration fracture, which can in turn result in restoration loss [6–8]. To reduce polymerization shrinkage and shrinkage stress many clinical methods have been suggested such as low-modulus intermediate resin liner application, soft-start light curing or pulse-delay curing as well as incremental layering techniques [9–12]. Clinical results of direct restorations with the incremental layering technique and curing systems mentioned above have shown positive results. Dentists have therefore used these for large cavities as a standard method. However, there are various disadvantages such as the potential for contamination or failures in bonding between layers and extended treatment time for material placement and polymerization [13].

The so-called flowable ‘bulk-fill’ composites with lower filler content have been brought to the market. Despite similar chemical composition as conventional flowable composites, it can be applied in bulks of 4–6 mm depending on the individual product due to enhanced polymerization depth. The simplified procedures make the bulk filling technique popular with clinicians and several advantageous outcomes were noted such as lower polymerization shrinkage and stress, reduced cusp deflection, and improved self-levelling ability compared to conventional flowable composites [14–16]. The low-viscosity bulk-fill composites, however, require capping with a conventional composite due to their inferior mechanical properties compared to conventional composites [17, 18]. To enable restorations from a single bulk-fill material, sculptable high-viscosity bulk-fill composites were developed to allow the filling of the whole cavity without any coverage. However, the flowable materials offer advantages due to their flow behavior during application in situations with complex cavity designs, as higher viscosity materials can complicate adaptation.

Interfacial adhesive defects (IAD; adhesive defects between composite restorations and tooth substance) and marginal adaptation seem to play a vital role in restoration durability. IAD and poor marginal adaptation can lead to plaque accumulation, discoloration, hypersensitivity, carious lesions, defect of restorations, or restoration loss [19]. A longitudinal assessment of both internal and marginal adhesive defects is needed to evaluate in detail the properties of these materials in clinical application [20, 21].

The current study was therefore designed to evaluate the marginal and interfacial gap formation when using two low-viscosity bulk-fill composites on the tooth surface in class II cavities. A conventional composite to be incrementally layered and a high-viscosity bulk-fill composite were used as control materials. As different adhesive systems might influence the quality of the interfacial/marginal adhesion, the bulk-fill composites were bonded with two different adhesive systems, namely an etch-and-rinse (ER) and a one-step self-etch adhesive (1-SE). Furthermore, the influence of artificial ageing on interfacial and marginal adaptation was evaluated. The null hypothesis was tested that no differences could be observed between all composites. Moreover, it was hypothesized that the ER adhesive would show an increased bonding-performance than the 1-SE adhesive and that artificial ageing has a detrimental influence on the integrity of the tooth-composite interface.

## Materials and methods

This blinded randomized controlled in vitro study was performed with two low-viscosity bulk-fill composites (SDR, x-tra base), each with two different adhesive systems (OptiBond FL, Xeno V+) (Table 1). These groups were compared to a layered nano-filled hybrid composite (Premise) and a high-viscosity bulk-fill composite (Tetric EvoCeram Bulk fill) with regard to the formation of marginal gaps (MG) and interfacial adhesive defects (IAD) [22]. 128 intact, non-carious, unrestored human molars were selected out of a pool of collected teeth.

**Table 1:** Materials under investigation and manufacturer information

Classification	Product name	Code	Manufacturer	Shade	Thickness max. (mm)	Resin matrix	Filler (w/v%)	LOT
Bulk-fill	SDR	SDR	DENTSPLY DeTrey Konstanz, Germany	U	4	Modified urethane dimethacrylate (UDMA), ethoxyliertes bisphenol-A-dimethacrylate (EBPADMA), triethylenglycol-dimetharcylat (TEGDMA)	Ba-Al-F-B silicate glass, Sr-A-F silicate glass (68/45)	1203000334
	x-tra base	XB	VOCO, Cuxhaven, Germany	U	4	Aliphatic UDMA, bisphenol A ethoxylate dimethacrylate (bis-EMA)	Inorganic filler in a methacrylate matrix (75/61)	1209632
	Tetric EvoCeram Bulk fill	TEC	Ivoclar Vivadent AG, Schaan, Liechtenstein	IVW	4	Bisphenol-A-glycidylmethacrylat (Bis-GMA), UDMA	Barium glass, ytterbium trifluoride, mixed oxide, prepolymer (81/61)	P84585
Incrementally layered	Premise	P	Kerr Corporation, Orange, CA, USA	A1	2.5	Ethoxylated bisphenol methacrylate (EBPADMA), diurethandimethacrylate (DUDMA), hexanediol dimethacrylate (HDDMA), hexamethylendiacrylate (HDDA), TEGDMA	PPF, Barium glass, silica fillers (84/70)	4451392
ER-adhesive	OptiBond FL	OFL	Kerr Corporation, Orange, CA, USA	–	–	Primer: 2-hydroxyethylmethacrylate (HEMA), glycerol phosphate-dimethacrylate (GPDM), phtalic acid monomethacrylate (MMEP)	–	4547701
						Adhesive: HEMA, trimethoxysilylpropyl methacrylate, glycerol dimethacrylate (GDMA)	Fumed SiO_2_, barium aluminoborosilicate, Na_2_SiF_6_ (48/–)	4516232
SE-adhesive	Xeno V+	X	DENTSPLY DeTrey Konstanz, Germany	–	–	Bifunctional acrylates, ethyl 2-[5-dihydrogen phosphoryl-5,2-dioxapentyl]acrylate, acidic acrylate	–	1112000699

### Restoration procedure

One operator (dentist) prepared all standardised MOD cavities with a rounded cylindrical diamond bur (012, 80 µm, APS, Intensiv SA, Grancia, Switzerland) under oil-free, extensive water cooling and refined the cavities with a cylindrical diamond finisher (012, 25 µm, APS, Intensiv SA). No margin was bevelled and all inner angles slightly rounded.

**Fig. 1:**
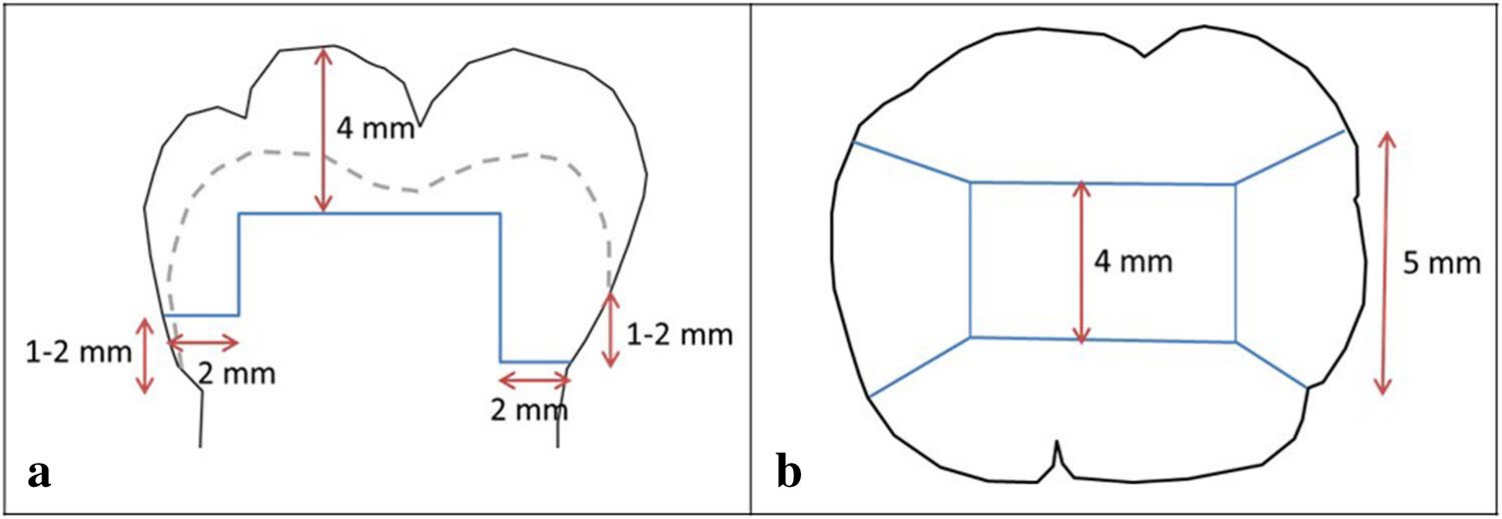
Extent of standardized class II cavity. **a** Cross sectional view (vestibular or lingual), **b** occlusal view. The dotted line indicates the enamel-dentin-junction/cemento-enamel junction

The dimensions of the class II cavities (Fig. 1) were (i) for the occlusal box: bucco-lingual 4 mm width, occlusal 4 mm depth; (ii) for the mesial box: mesio-distal 2 mm and bucco-lingual 5 mm width, the mesial margin was located 1–2 mm above the cemento-enamel junction (5 ± 1 mm below the peak of protuberance); (iii) distal box: mesio-distal 2 mm and bucco-lingual 5 mm width, the distal margin was positioned 1–2 mm below the cemento-enamel junction (7 ± 1 mm below the peak of protuberance). After preparation, all cavities were rinsed with water and the molars were stored in a climate chamber for a maximum of 1 h [22].

All restorations were performed according to the manufacturer’s instructions by one operator, who had practised the handling of the materials several times before starting this study. Table 1 shows the properties and composition of the materials used. Table 2 summarizes the procedures for application. The two low-viscosity bulk-fill composites were covered occlusally with an at least 2 mm thick surface layer of the nano-filled-hybrid composite Premise. The control restorations were placed with Premise in conventional 2 mm increments. After restoration, overhangs were initially removed with a scaler (S204SD9, Hu-Friedy, Leimen, Germany). The margins were finished proximally with a bud-shaped diamond finisher (15 µm, 20,000 min^−1^; APS, Intensiv SA) and occlusally with a grenade-shaped diamond finisher (9 µm, 20,000 min^−1^, APS) as well as polished (4000 min^−1^; Politip-F grey, Ivoclar Vivadent AG, Schaan, Principality of Liechtenstein). Afterwards, all specimens were extensively rinsed and stored in water for 24 h (pure water ASTM III, 37 °C; Micromed 6, TKA; Niederelbert, Germany).

**Table 2:** Procedures for application of used restorative materials according to manufacturer’s recommendation

Material	Workflow
Adhesive	
OptiBond FL	1. Etching with 37.5% H_3_PO_4_ (30–40 s for enamel, 15–20 s for dentin) 2. Adaptation of matrix (KerrHawe Lucifix^®^ Molar Bands Transparent, KerrHawe SA, Bioggio, CH) 3. Application of Primer for 15 s 4. Evaporating the solvent using oil-free air for at least 5 s 5. Application of adhesive for 15 s 6. Blowing for 3 s 7. Light-cure for 20 s with direct tooth contact (1100 mW/cm^2^ ± 10%; bluephase (G2), Ivoclar Vivadent AG, Schaan, Liechtenstein)
Xeno V+	1. Adaptation of Matrix (KerrHawe Lucifix^®^ Molar Bands Transparent) 2. Application of self-etch adhesive for 20 s 3. Blowing for min. 5 s 4. Light-cure for 10 s with direct tooth contact (1100 mW/cm^2^ ± 10%; bluephase)
Composite	
SDR	1. Inserting the cap into the dispenser 2. Application of the filling material onto the cavity (layers of 4 mm thickness max.) 3. Light cure from the occlusal, vestibular and oral surface for 20 s each with direct tooth contact (1100 mW/cm^2^ ± 10%; bluephase) 4. Application of the Premise (nano-filled hybrid composite, reference composite) onto the cavity (layers of 2 mm thickness min.) as an occlusal coverage 5. Light-cure from the occlusal, vestibular and oral surface for 10 s each with direct tooth contact (1100 mW/cm^2^ ± 10%; bluephase)
x-tra base	1. Inserting the cap into the dispenser 2. Application of the filling material into the cavity at the deepest point (layers of 4 mm thickness max.) 3. Light cure from the occlusal, vestibular and oral surface for 10 s each with direct tooth contact (1100 mW/cm^2^ ± 10%; bluephase) 4. Application of the Premise (nano-filled hybrid composite, reference composite) onto the cavity (layers of 2 mm thickness min.) as an occlusal coverage 5. Light-cure from the occlusal, vestibular and oral surface for 10 s each with direct tooth contact (1100 mW/cm^2^ ± 10%; bluephase)
Tetric EvoCeram Bulk Fill	1. Inserting the cavifil into the injector 2. Application of the filling material onto the cavity (layers of 4 mm thickness max.) 3. Build-up with modellation instruments 4. Light-cure from occlusal, vestibular and oral surface for 10 s each with direct tooth contact (1100 mW/cm^2^ ± 10%; bluephase)
Premise	1. Inserting the unidose into the dispenser 2. Application of the filling material onto the cavity (layers of 2.5 mm thickness max.) 3. Build-up with modellation instruments 4.Light-cure from occlusal, vestibular and oral surface for 10 s each with direct tooth contact (1100 mW/cm^2^ ± 10%; bluephase)

### Artificial ageing

All specimens were divided into two groups (*n* = 8; with artificial loading, without artificial loading). Before artificial ageing impressions of the filling margins were taken (Coltène PRESIDENT putty soft and plus light body, Coltène/Whaledent AG, Altstätten, Switzerland). The specimens were stored in water for 180 days (37 °C, water change 2 × per week) and following thermocycled 2500 times (TC; 5–55 °C, 1 min per cycle; Willytec Thermocycler V2.8, Feldkirchen-Westerham, Germany). After the artificial ageing, a new set of impressions was obtained. With all impressions, epoxy resin replicas (Stycast 1266 Part A + B, Emerson and Cumming, Westerlo, Belgium) were made for the imaging and analysis of the marginal gaps before and after artificial ageing [22].

### Analysis of marginal and internal adaptation

For the MG analysis, all replicas were mounted on aluminium specimen stubs (12.5 mm Ø, Plano GmbH, Wetzlar, Germany), sputter-coated (5 nm, Edwards Sputter Coater S150B, BOC Edwards, Irvine, Great Britain) and examined with scanning electron microscopy (SEM, 200×; Phenom G2 Pro, Phenom-World BV, Eindhoven, The Netherlands). MG between resin composite and enamel or dentin were expressed as a percentage of the full margin length of enamel or dentin, respectively.

For the analysis of IAD all specimens were rinsed (15 s, 20 °C) and stored in 5% glutaric di-aldehyde in 0.1 M sodium phosphate buffer (pH 7.2; 24 h, 4 °C). After rinsing three times again with 0.1 M sodium phosphate buffer (pH 7.2; 1 × per h, 20 °C), the specimens were embedded in epoxy resin (Stycast 1266 Part A + B, Emerson & Cuming, Westerlo, Belgium) and sectioned longitudinally (five slices per specimen, 200 µm) using a microtome (Leitz 1600 sawing-microtome, Ernst Leitz Wetzlar GmbH, Wetzlar, Germany). The second and the fourth slice per specimens were selected for the direct illustration of internal adaptation. The slices were etched with HCl (2%, 10 s, 20 °C) and NaOCl (10%, 30 s, 20 °C) and rinsed with pure water (60 s, 20 °C) after every step. Followed by careful dehydration using the chemical effect of an afferent alcohol chain (30–50–70–80–90–95–100–100–100%) and hexamethyldisilazane (10 min, 20 °C, HMDS, Carl Roth GmbH, Karlsruhe, Germany). After gentle air-drying, each slice was placed on an aluminium specimen stub (12.5 mm Ø, Plano GmbH, Wetzlar, Germany) and gold-coated (5 nm) in a sputtering device (Edwards Sputter Coater S150B, BOC Edwards, Irvine, Great Britain). The specimens were examined with scanning electron microscopy (200x) and IAD were scored by one operator in relation to the total interface length based on the following intervals: score 1: 0–25%, score 2: > 25–50%, score 3: > 50–75%, score 4: > 75–100% [22].

### Statistical analysis

Assuming a power of 70%, sample size calculation (G*Power 3.1.9.2, free, Heinrich-Heine-University Düsseldorf) resulted in *n* = 5 specimens per group for MG and *n* = 7 per group for IAD. In each group, *n* = 8 specimens were used. Whereas the operator applying the restorations was aware of the allocation to the adhesive materials, the examiners and the data analysts were kept blinded to the allocation. The endpoints of analysis were the length of internal and marginal gap formation without/before and with/after artificial loading. SPSS 20.0 for Windows (SPSS Inc., Chicago, IL, USA) was used to analyse the data. Kruskal–Wallis and Mann–Whitney *U* test as well Friedman- and Wilcoxon-test were used for comparison of the groups (*α* = 0.05). Due to the exploratory nature of this research, raw *p* values are reported and we refrained from correction for multiple testing.

## Results

Regardless of adhesive system and artificial ageing, there were no significant differences between composite types in 44 out of 48 comparisons for MG (92%, *p* ≥ 0.077) or 43 out of 48 comparisons for IAD (90%, *p* > 0.05). The groups in which the composites were applied with X showed significantly more IAD in 14 of 16 comparisons with OFL while the composites with X demonstrated more MG in 13 of 16 comparisons (significant: 2 comparisons). After artificial ageing, MG generally increased (significant: 9 out of 16) and IAD increased in 10 out of 16 groups (significant: 1).

### Marginal gap formation (Table 3)

**Table 3:** Marginal gap formation (%) at approximal cavity outline at enamel and dentin before/after artificial ageing

Composite	Adhesive	Enamel		Dentin	
		Before artificial ageing	After artificial ageing	Before artificial ageing	After artificial ageing
SDR	Optibond FL	5.6 ± 5.7^A,C^	24.5 ± 10.7^C^	0.8 ± 1.8^a^	21.9 ± 24.1^a^
	Xeno V+	19.4 ± 10.0^A,D^	38.1 ± 18.1^D,H^	7.2 ± 7.5	22.0 ± 22.6
x-tra base	Optibond FL	15.4 ± 9.4^E^	31.5 ± 19.8^E^	3.6 ± 4.3^b^	37.4 ± 27.5^b,e^
	Xeno V+	26.1 ± 9.7^F^	54.8 ± 8.9^F^	17.5 ± 23.2^c^	60.0 ± 22.0^c,f^
Tetric EvoCeram BF	Optibond FL	9.3 ± 7.1	15.7 ± 10.6	8.9 ± 9.2	20.0 ± 21.8
	Xeno V+	27.8 ± 11.8	30.5 ± 11.3^H^	5.5 ± 6.1	12.1 ± 12.9^f,g^
Premise	Optibond FL	6.6 ± 2.6^B,G^	21.6 ± 7.9^G^	6.2 ± 10.0	9.6 ± 16.3^e^
	Xeno V+	25.6 ± 13.0^B^	33.4 ± 19.9	5.1 ± 3.0^d^	26.3 ± 17.1^d,g^

Means followed by the same superscript letters are significantly different (*p* < 0.05)

At enamel: All composites in combination with OFL showed no differences among each other (*p* > 0.05). In combination with X, SDR showed more MG than TEC after artificial ageing (*p* < 0.05). At dentin: While in group XB/OFL after artificial ageing more MG were induced compared to P/OFL, group TEC/X showed less MG than XB/X and P/X (*p* < 0.05). The combination of SDR/OFL, XB/OFL, XB/X and P/X showed significantly more MG after artificial ageing (*p* < 0.05).

Enamel and dentin: 14 out of 16 pairwise comparisons (81%) showed no significant differences between both adhesive systems and 7 out of 16 pairwise comparisons (44%) showed no significant difference between before and after artificial ageing.

### Internal adhesive defects (Table 4; Fig. 2)

**Table 4:** Adhesive defects at the enamel/dentin-composite interfaces (mean score)

Composite	Adhesive	Enamel		Dentin	
		Without artificial ageing	With artificial ageing	Without artificial ageing	With artificial ageing
SDR	Optibond FL	1.3 ± 0.6^A^	1.6 ± 0.8	1.1 ± 0.2^a^	1.4 ± 0.4^b^
	Xeno V+	2.5 ± 1.4^A^	2.2 ± 1.2^H,I^	3.2 ± 0.8^a,i,j^	3.4 ± 0.7^b^
x-tra base	Optibond FL	1.3 ± 0.6^B^	2.1 ± 1.2^C^	1.3 ± 0.6^c^	1.2 ± 0.5^d^
	Xeno V+	3.7 ± 0.8^B^	3.9 ± 0.3^C,H^	4.0 ± 0.0^c,i^	3.9 ± 0.2^d^
Tetric EvoCeram BF	Optibond FL	1.0 ± 0.0^D,J^	1.0 ± 0.0^E^	1.0 ± 0.0^e^	1.1 ± 0.3^f^
	Xeno V+	3.1 ± 1.0^D^	3.8 ± 0.5^E,I^	4.0 ± 0.0^e,j^	3.8 ± 0.5^f^
Premise	Optibond FL	2.0 ± 1.0^F,J^	1.0 ± 0.0^F,G^	1.3 ± 0.4^g^	1.7 ± 1.0^h^
	Xeno V+	2.8 ± 1.4	3 ± 1.3^G^	3.4 ± 0.7^g^	3.7 ± 0.5^h^

Means followed by the same superscript letters are significantly different (*p* < 0.05)

**Fig. 2:**
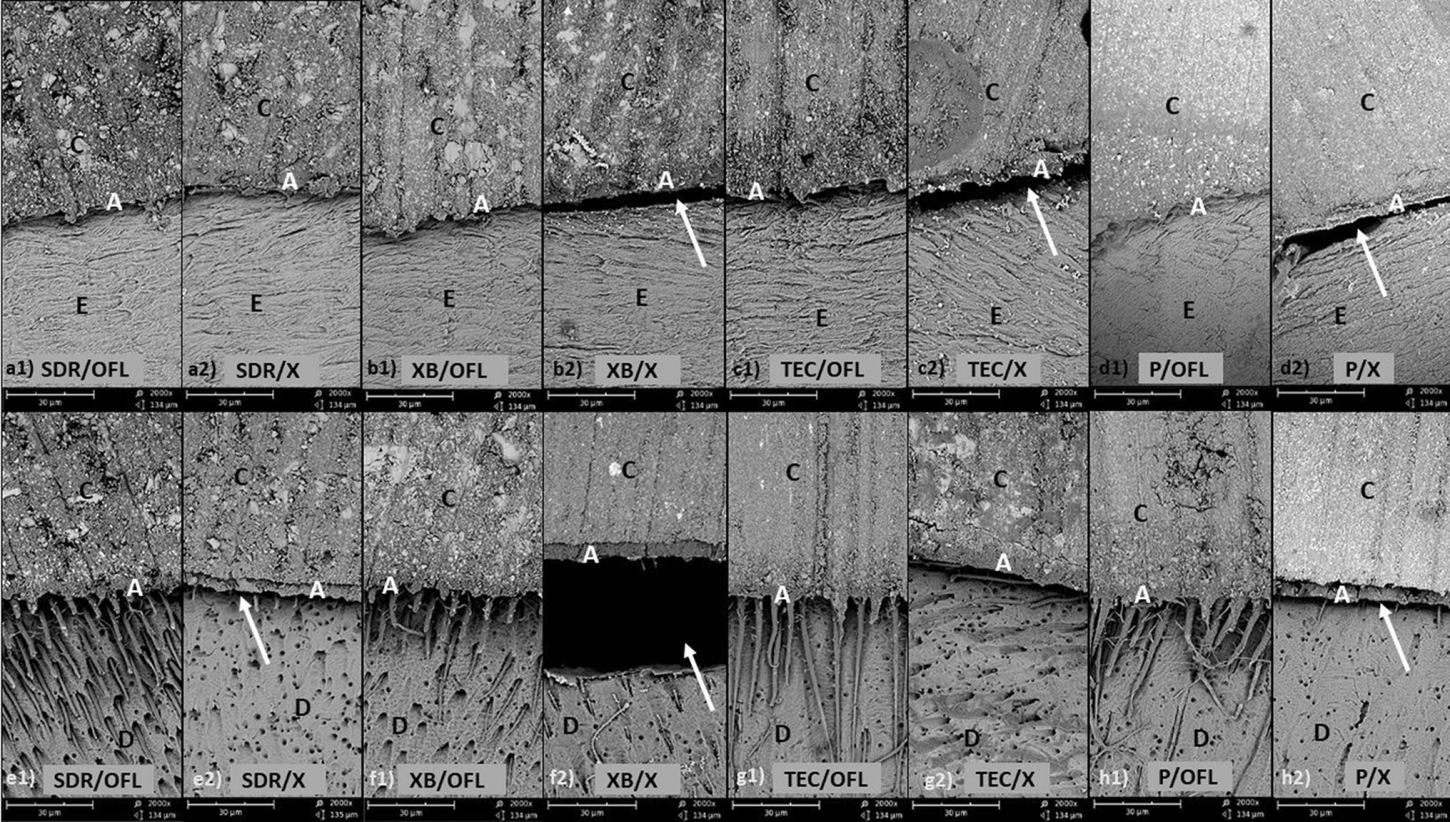
SEM images (×200 magnification). a–d enamel-composite interfacial zone; e–h dentin-composite interfacial zone; at enamel, adhesive defects were observed in the groups with X. At dentin all groups with OFL, with the exception of XB, showed distinct resin tags with deep penetration; white arrows: adhesive defects, *C* composite, *E* enamel, *D* dentin

At enamel: Except that TEC had less IAD than *p* without artificial ageing (*p* < 0.05), all other composites in combination with OFL regardless of artificial ageing showed no differences among each other (*p* ≥ 0.077). In combination with X, SDR with artificial ageing showed less IAD than XB and TEC (*p* ≤ 0.035). Premise in combination with OFL showed significantly more IAD with artificial ageing (*p* < 0.05). At dentin: All composites in combination with OFL regardless of artificial ageing showed no differences among themselves (*p* ≥ 0.282). In combination with X, SDR without artificial ageing showed less IAD than XB and TEC (*p* < 0.05). No significant differences between specimens without artificial ageing and these with artificial ageing were observed (*p* > 0.07). Fourteen out of 16 pairwise comparisons (88%) indicated that the composites with X showed more IAD than those with OFL. With the exception of the restoration system P/OFL for all other systems (bulk-fill composites), no influences of artificial ageing on IAD was observed at enamel and dentin regardless of the adhesive system (*p* > 0.05).

## Discussion

Successful composite restoration depends on many factors such as mechanical properties of materials and the integrity of the composite bond to tooth, which is determined by the quality of the operation [23]. The mechanical durability of composites, bonding performance of adhesives and handling of materials have constantly improved over the last decade [23]. Nevertheless, the integrity- and sealing ability of composite materials to a tooth is still a considerable challenge. Poor adaption of composite to tooth can lead to microleakage, discoloration, restoration fracture and consequently, loss of restoration [13]. The integrity of the tooth-composite bond is influenced by many factors such as degree of conversion of the composite, volumetric polymerization contraction, polymerization shrinkage stress as well as the interaction between composites, adhesives and tooth hard substances [8, 24]

The null hypothesis of this study cannot be rejected as more than 90% pairwise comparisons showed no differences among all composites regardless of adhesive type and artificial ageing. The degree of conversion (DC) of the composites in depth of 4 mm investigated in the current study varies from 58.6% (XB) to 76.1% (SDR) [25–28]. Although DC of XB is clinically acceptable (> 55%), XB showed significantly lower DC than other conventional and bulk-fill composites [25]. Previous studies [29, 30] reported that the volumetric polymerization shrinkage of the composites investigated are 2.02% (P), 2.03% (TEC), 2.76% (SDR) and 2.8% (XB). The volumetric polymerization shrinkage of both reference composites TEC and *p* are significantly lower than the low-viscosity bulk-fill composites. It is well known that low-viscosity composites have higher polymerization contraction due to lower filler volume compared to high-viscosity composites. The high-viscosity composites generally have a higher elastic modulus, which positively correlates with the polymerization shrinkage stress induction and in turn, poor integrity of the composite to teeth [31]. This fact generally also applies for high-viscosity bulk-fill composites, except TEC. TEC has a lower elastic modulus compared to that of the conventional high-viscosity composite as TEC contains pre-polymerized filler and consequently a lower fraction of inorganic filler, which contributes to the increase of the elastic modulus [32]. Based on this advantage of TEC it is expected that TEC shows similar adhesive performance as the low-viscosity bulk-fill composites (SDR, XB) which is in line with the results of the current study. On the other hand, an improved adhesive performance compared to the conventional high-viscosity composite (*P*) could be expected which however was not observed in the current study. The time frame of the experiment may have been too short to recognize significant effects.

Numerous studies confirmed that the polymerization shrinkage stress of SDR is lower than other low-viscosity (conventional and bulk-fill) composites or conventional high-viscosity composites [15, 33–35]. At the significance level of *α* = 0.05, 43 of 48 group comparisons in the current study showed no significant differences between SDR and the other composites in marginal and internal adaption, independent of the type of adhesive and artificial ageing, except for particular cases (SDR/X vs. XB/X). Many studies reported similar margin sealing ability of bulk-fill composites and conventional composites [30, 36–42], which is comparable with the results of the current study. Agarwal et al. concluded that low-viscosity bulk-fill composites showed an enhanced performance with regard to internal adaptation than high-viscosity bulk-fill composites [43]. However, the current study has shown that sealing ability is product-specific and this statement does not apply to this study.

The hypothesis that the ER adhesive would perform more effectively than the 1-SE adhesive, is mostly confirmed for the parameter “interfacial adhesive defect”. Different adhesive systems with bulk-fill composites showed apparent effects on adhesive defects regardless of the marginal tooth substrate investigated (enamel or dentin) but non-significant effects on marginal gap formation. A tendency of increased marginal gap formation with the 1-SE was observed. This could result from the small sample size combined with the sample scattering, the large scatter of the measured values (standard deviation) and the susceptibility to errors of the adhesive application process. A further explanation might be that the reduced polymerization shrinkage stress of bulk-fill composites positively influences the marginal adaptation of poorly performing adhesive systems such as 1-step SE. It was reported that ER adhesive systems showed generally enhanced adhesive performance especially than 1-SE adhesive systems in combination with the conventional composites [44, 45]. Furthermore, filled adhesives (OFL) are generally expected to achieve a more stable bond than unfilled adhesives (X), as they act as an “elastic buffer” and prevent excessive thinning of the adhesive layer [46]. Additionally, filled adhesives show higher stability because the initial polymerization of the adhesive layer cannot be completely inhibited by oxygen as is the case with very thin layers [47]. Concerning bulk-fill composites, Al-Harbi et al. presented no significant differences for the criterion “perfect margin” between ER and SE (1-step and 2-step) [36], which is in line with the results of the current study. Roggendorf et al. indicated a significantly higher percentage of continuous margins with ER at dentin than 1-step SE [42], while Takahashi et al. found significantly better marginal adaptation with SE (1-step and 2-step) than ER [48]. This variance might result from the study designs, sub-classification of adhesives and product specific adhesion ability within the same adhesive classification.

The hypothesis that artificial ageing has a detrimental influence on the integrity of composites to tooth, cannot be generally accepted. It was expected that marginal gap formation and interfacial adhesive defect increase after artificial ageing. This applies, in particular, in both groups with a combination of X (4×) and OFL (5×) due to higher degradation [49] and hydrolysis of the hydrophilic resin [50]. The current study simulated ageing by thermocycling and water storage and there was a 56% significant influence of artificial ageing on marginal gap formation. The reason for this could be the brevity of the investigation of less than 1 year with a moderate amount of thermal cycles [51]. It is reported that approximately 10,000 thermal cycles equate to 1 year of clinical service [52]. Additionally, water absorption of composites during storage and thermocycling may work as compensation for the impact of the polymerization shrinkage/shrinkage stress at the initial phase [53]. The fact that the increase of marginal gap formation/interfacial adhesive defect is often not significant, resulted from a number of critical aspects inherent to the study’s method already discussed above (sample size, scatter).

In the current study, the interfacial adhesive defects were evaluated by SEM analysis on two cross sections per sample. False positive results can occur as a result of sample preparation such as sectioning and dehydating of samples [54]. In contrast to the internal adaptation, the marginal integrity was measured by a metric parameter and non-invasively by quantitative margin analysis using replicas so that the same specimens were evaluated before and after artificial ageing, resulting in a higher statistical power of the examination. Although both methods provide limited information about the three-dimentional geometry [54], they reveal adhesive defects indicating a lack of chemical bonding of the restoration. Notwithstanding these methodical limitations, the strengths of this study setup are that both marginal- and internal adaptation of composite were evaluated taking into consideration the complexity of the adhesive performance of composite restorations in class II cavities. Additionally, important factors such as adhesive type and artificial ageing were evaluated.

The low-viscosity bulk-fill composites showed lower macro- and micromechanical properties (elastic modulus, elastic indentation, and Vickers hardness) than high-viscosity bulk-fill composites [55]. However, for the low-viscosity composites, the Weibull-parameters indicate high material reliability represented by a high homogeneity and defect-free material, which among other things may indicate a simpler and more reliable application [32]. Additionally, the current study showed no detrimental effects on the tooth-composite integrity of the low-viscosity composites. Therefore, the low-viscosity bulk-fill composites used can be considered for the restoration of class II cavities if the flowability offers advantages in handling. At the same time, bulk-fill composites in combination with the 1-step self-etch adhesive Xeno V+ are not recommended to be used for restoration of class II cavities.

## Conclusion

The performance of the investigated composite types with regard to the integrity of the tooth-composites interface was comparable. The etch-and-rinse adhesive used was superior to the 1-step self-etch system for internal adaptation and artificial ageing has a detrimental influence on marginal adaptation regardless of the adhesive and composite type.
