# Correction to: Evaluation of low-viscosity bulk-fill composites regarding marginal and internal adaptation

**DOI:** 10.1007/s10266-021-00666-5

**Published:** 2021-10-19

**Authors:** Kyung-Jin Park, Manon Pfeffer, Thomas Näke, Hartmut Schneider, Dirk Ziebolz, Rainer Haak

**Affiliations:** Department of Cariology, Endodontology and Periodontology, University of Leipzig, Liebigstr.12, 04103 Leipzig, Germany

## Correction to: Odontology (2021) 109:139–148 10.1007/s10266-020-00531-x

The article Evaluation of low-viscosity bulk-fill composites regarding marginal and internal adaptation, written by Kyung Jin Park, Manon Pfefer, Thomas Näke, Hartmut Schneider, Dirk Ziebolz and Rainer Haak, was originally published Online First without Open Access. After publication in volume 109, issue 1, pages 139–148 the author decided to opt for Open Choice and to make the article an Open Access publication. Therefore, the copyright of the article has been changed to © The Author(s) 2021 and the article is forthwith distributed under the terms of the Creative Commons Attribution 4.0 International License, which permits use, sharing, adaptation, distribution and reproduction in any medium or format, as long as you give appropriate credit to the original author(s) and the source, provide a link to the Creative Commons license, and indicate if changes were made. The images or other third party material in this article are included in the article’s Creative Commons licence, unless indicated otherwise in a credit line to the material. If material is not included in the article’s Creative Commons licence and your intended use is not permitted by statutory regulation or exceeds the permitted use, you will need to obtain permission directly from the copyright holder. To view a copy of this licence, visit http://creativecommons.org/licenses/by/4.0. Open access funding enabled and organized by Projekt DEAL.

The original article has been updated.

